# An Intelligent System for Identifying Acetylated Lysine on Histones and Nonhistone Proteins

**DOI:** 10.1155/2014/528650

**Published:** 2014-07-24

**Authors:** Cheng-Tsung Lu, Tzong-Yi Lee, Yu-Ju Chen, Yi-Ju Chen

**Affiliations:** ^1^Department of Computer Science and Engineering, Yuan Ze University, Taoyuan 320, Taiwan; ^2^Institute of Chemistry, Academia Sinica, Taipei 115, Taiwan

## Abstract

Lysine acetylation is an important and ubiquitous posttranslational modification conserved in prokaryotes and eukaryotes. This process, which is dynamically and temporally regulated by histone acetyltransferases and deacetylases, is crucial for numerous essential biological processes such as transcriptional regulation, cellular signaling, and stress response. Since the experimental identification of lysine acetylation sites within proteins is time-consuming and laboratory-intensive, several computational approaches have been developed to identify candidates for experimental validation. In this work, acetylated protein data collected from UniProtKB were categorized into histone or nonhistone proteins. Support vector machines (SVMs) were applied to build predictive models by using amino acid pair composition (AAPC) as a feature in a histone model. We combined BLOSUM62 and AAPC features in a nonhistone model. Furthermore, using maximal dependence decomposition (MDD) clustering can enhance the performance of the model on a fivefold cross-validation evaluation to yield a sensitivity of 0.863, specificity of 0.885, accuracy of 0.880, and MCC of 0.706. Additionally, the proposed method is evaluated using independent test sets resulting in a predictive accuracy of 74%. This indicates that the performance of our method is comparable with that of other acetylation prediction methods.

## 1. Introduction

Lysine acetylation is a dynamic and reversible posttranslational modification (PTM) that is highly conserved in prokaryotes and eukaryotes. This process neutralizes the positive charge on an amino acid and regulates DNA binding, protein-protein interaction, and protein stability [1, 2]. Lysine acetylation conjugates on *ε*-amino group of lysine residues and is regulated by a highly balanced enzyme system containing lysine acetyltransferases (KATs, also known as histone acetyltransferases HATs) and histone deacetylase (HDACs). Furthermore, acetylation also occurs on *α*-amino groups of N-terminal residues (N^*α*^-lysine acetylation) [1, 3]. In addition, lysine acetylation is involved in diverse biological consequences including transcriptional activity, cell survival, and subcellular localization [4–7]. Most importantly, it has been reported that aberrant lysine acetylation is linked to many pathological diseases, such as cancer, neurodegenerative diseases, and metabolic diseases [8–12]. Following the identification of nuclear KATs, a number of nonhistone proteins have been identified as substrates, including DNA-binding proteins (transcription factors), nonnuclear proteins, and shuttle proteins from the nucleus to cytoplasm [1, 13, 14]. To date, over 2000 human acetylated proteins and 4000 lysine sites have been identified by traditional experiments and large-scale mass spectrometry-based proteomic analysis [15]; however, determining the acetylation substrate specificity of KATs remains a challenge. Moreover, KATs responsible for nonhistone proteins are still unclear.

Previous efforts on this area focus on the determination of acetylation sites using conventional experiments and mass spectrometry. These approaches are often time intensive, expensive, and laborious. Using a birelative adapted binomial score Bayes (BRABSB) and a support vector machine (SVM), Shao et al. have developed a predictor for human lysine acetylation from short linear motifs catalyzed by acetyltransferases and captured linear/nonlinear correlation among residues around target lysine residues [16]. Another tool, N-Ace, uses data clustering with specific subcellular localization (e.g., histones, nucleus, cytoplasm, membrane, and mitochondrion) to distinguish the potential substrates catalyzed by different localization of specific acetyltransferases [17]. Several computational tools for predicting acetylation sites have been developed, such as NetAcet [18], PAIL [19], LysAcet [20], and EnsemblePAIL [21]. However, the characteristic of lysine acetylation between histone and nonhistone proteins is still unclear.

In this study, we describe the features and capabilities of AceK—a tool for identifying lysine acetylation on histone and nonhistone proteins. Using a clustering method via maximal dependence decomposition (MDD) [22], a large group of aligned sequences is moderated into subgroups that capture the most significant dependencies between positions. Further evaluation was done, using fivefold cross-validation, which shows that the SVM models trained with MDD-clustered subgroups exhibit an improved predictive accuracy as compared to the non-MDD clustered models. Based on the output of the model, we describe the different characteristics and compositional biases of amino acids around acetylation sites and nonacetylation sites on histone and nonhistone proteins.

## 2. Material and Methods

### 2.1. Data Collection and Preprocessing

Protein sequences were obtained from UniProtKB/SwissProt (v5715) disregarding those without experimental evidence which are annotated as “by similarity,” “potential,” or “probable” in the “MOD_RES” fields. From the collected data, lysine acetylated sites were used as the positive data of training set, while nonacetylated lysines were used as the negative data of training set, respectively. A total of 200 and 3325 acetylation sites on histone and nonhistone proteins, respectively, were obtained as positive data (Table 1). On the other hand, 2729 and 72445 nonacetylation sites on histone and nonhistone proteins, respectively, were obtained as negative data. In order to avoid a biased prediction performance for a binary classification between positive and negative data, the negative training data was balanced with the positive training data. A *K*-means clustering method based on sequence identity [23, 24] was employed for acquiring a subset that represented the whole negative dataset. The number of corresponding positive data was set as the value of *K* to denote an equal number of samples to be obtained from the negative set.

To form the independent testing set, experimentally verified acetylation sites were extracted from another version of UniProtKB/SwissProt (v2014_01) disregarding data existing in v5715 of the same database. A total of 9 and 66 acetylation sites and 93 and 1737 nonacetylation sites from histone and nonhistone proteins, respectively, were obtained for the independent testing dataset. After the cross-validation of training set, the independent test set was evaluated by using the trained model with the highest accuracy. However, the positive data of independent test set may include the sequences that were homologous to training data. As for classification, the prediction performance of the trained models may be overestimated owing to the overfitting of a training set. To prevent an overestimation in the predictive performance, homologous sequences between training set and independent test set were removed. With reference to the reduction of the homology of the training set in MASA [23], two acetylated protein sequences with more than 30% identity were defined as homologous sequences. Then, two homologous sequences were specified to realign the fragment sequences using a window length of 2*n* + 1, centered on the acetylation sites using BL2SEQ [25]. For two fragment sequences with 100% identity, the acetylation site on the homologue fragment sequence in the testing set was discarded, leaving only the acetylation site on the training set. Redundancy was also removed by retaining only one record in the event of finding multiple records of the same site position and accession number. Nonredundant negative data were generated using the same approach. After the removal of redundant data, 75 positive sequence fragments and 1830 negative sequence fragments with cysteine residues were obtained for independent testing.

### 2.2. Detection of Substrate Motif

**Figure 1:**
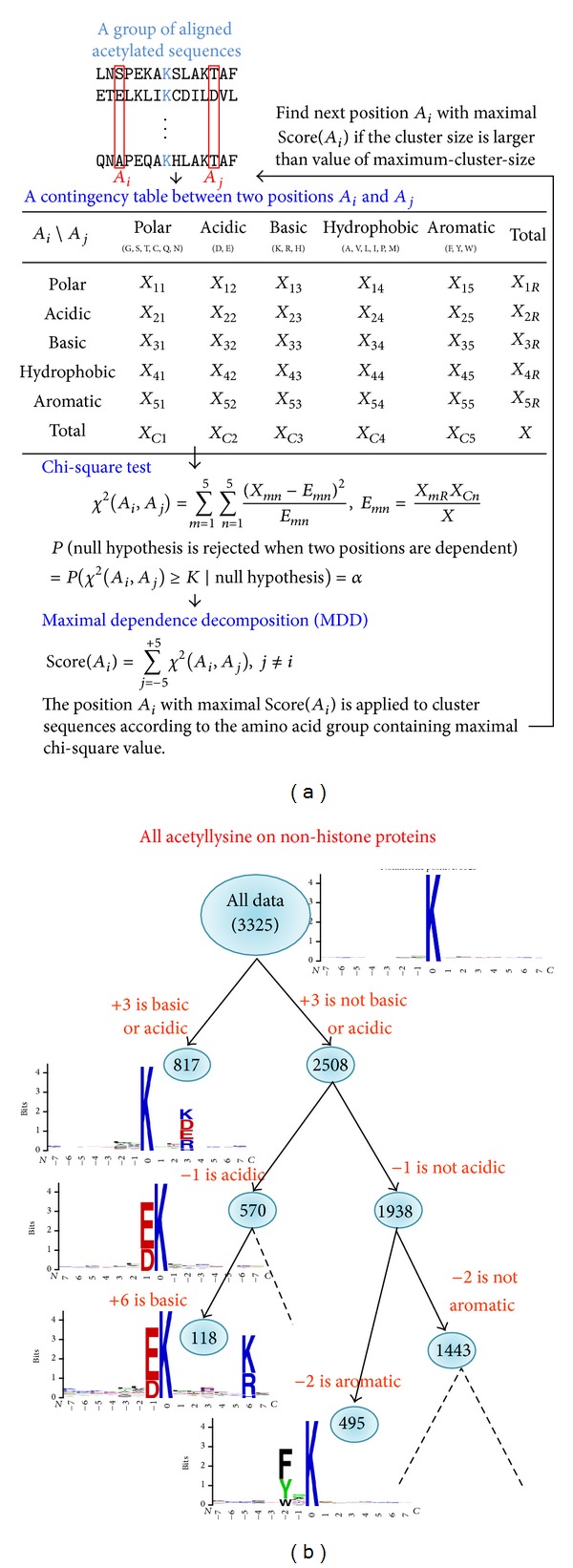
The analytical flowchart of MDDLogo application. Panel (a) shows that the process of a contingency table is created and used together with a chi-square test to obtain a maximal score for each position in a sequence. Panel (b) presents a tree-like visualization of MDDLogo clustering on a set of sequences.

One of the aims of this study is to investigate the motifs of acetylation sites based on amino acid sequences. Due to the difficulty of detecting conserved motifs for large sets of sequences, we applied maximal dependence decomposition (MDD) [22] to cluster all sequences of acetylation site into subgroups containing statistically conserved motifs. MDDLogo, a tool implementing MDD, has reported that the grouping of protein sequences into smaller groups should be done prior to computationally identifying PTM sites [26–32]. MDDLogo adopts a chi-square test *χ* ^2^(*A*_*i*_, *A*_*j*_) to evaluate the dependence of amino acid occurrence between two positions *A*_*i*_ and *A*_*j*_ surrounding acetylation lysines. In order to extract motifs containing conserved biochemical properties of amino acids, the 20 types of amino acids were categorized into five groups: polar, acidic, basic, hydrophobic, and aromatic groups (Table 2). A contingency table of amino acid occurrence between two positions was then constructed (Figure 1(a)). The chi-square test was defined as follows:

(1) $$\begin{array}{c} \chi^{2}\left(A_{i},A_{j}\right)=\overset{5}{\underset{m=1}{\sum }}\;\overset{5}{\underset{n=1}{\sum }}\frac{{\left(X_{mn}-E_{mn}\right)}^{2}}{E_{mn}}, \end{array}$$

where *X*_*mn*_ represents the number of sequences containing amino acids of group *m* in position *A*_*i*_ and amino acids of group *n* in position *A*_*j*_, for each pair (*A*_*i*_, *A*_*j*_) with *i* ≠ *j*. *E*_*mn*_ was calculated as (*X*_*mR*_ · *X*_*Cn*_)/*X*, where *X*_*mR*_ = *X*_*m*1_ + ⋯+*X*_*m*5_, *X*_*Cn*_ = *X*_1*n*_ + ⋯+*X*_5*n*_, and *X* denotes the total number of sequences. If a strong dependence is detected (defined as the value of *χ* ^2^ larger than 34.3, corresponding to a cutoff level of *P* ≤ 0.01 with 16 degrees of freedom) between two positions, then we proceeded as described by Burge and Karlin [22]. As illustrated in Figure 1(b), maximal dependence with the occurrence of basic amino acids was observed at position +3. Subsequently, all data can be divided into two subgroups: one with the occurrence of basic amino acids in position +3 and the other without the occurrence of basic amino acids in position +3. MDD clustering is a recursive process to divide the positive set into tree-like subgroups. When applying MDDLogo to cluster the sequences of a positive set, a parameter, that is, the maximum-cluster-size, should be set. If the size of a subgroup was less than the maximum-cluster-size, the subgroup will not be divided any more. In order to obtain an optimal minimum cluster size, MDDLogo was executed using various values. For this investigation, each subgroup resulting from MDDLogo was represented using WebLogo [33] for determining if they presented conserved motifs for the substrate specificity of acetylation or not.

### 2.3. Feature Extraction and Encoding

Support vector machine (SVM) was applied to develop the prediction models for identifying lysine methylation sites on histones and nonhistone proteins, respectively [34]. Seven features were taken into consideration into SVM models for a fragment sequence, including 20D binary code (AA), BLOSUM62 matrix (B62), amino acid composition (AAC), amino acid pair composition (AAPC), accessible surface area (ASA), position weight matrix (PWM), and position specific scoring matrix (PSSM). The 20D binary code (AA) is the most popular coding method using orthogonal binary coding scheme to transform each amino acid into 20-dimensional binary vector. Here we added a vector to represent other specific amino acid codes (e.g., *B*, *Z*, and *X*). So the number of the feature vector is 21∗*L*, where *L* is the window length of the fragment sequence. Amino acid composition (AAC) was obtained by converting a protein sequence into a 20-dimensional feature vector, where each vector consists of the composition of frequency of each of the twenty amino acids. Amino acid pair composition (AAPC) was obtained by converting a protein sequence from the frequency of amino acid pairs. Each amino acid coordinates with its adjacent residue, and therefore it can be transformed as a vector with 400 (20∗20) dimensions. Accessible surface area (ASA) refers to the surface area of a biomolecule that is accessible to a solvent, as a way for quantifying hydrophobic burial. Using the blocks substitution matrix (BLOSUM62), the substitution scores were derived from the alignments of amino acid sequences that had no more than 62% identity. Position specific scoring matrix (PSSM) refers to a matrix of score values generated from PSI-BLAST, which can represent the multiple sequence alignment of proteins. The scores are shown as positive or negative integers, and large positive scores often indicate critical functional residues, which may be the active site residues or residues required for other intermolecular interactions.

### 2.4. Model Construction and Cross-Validation

**Figure 2:**
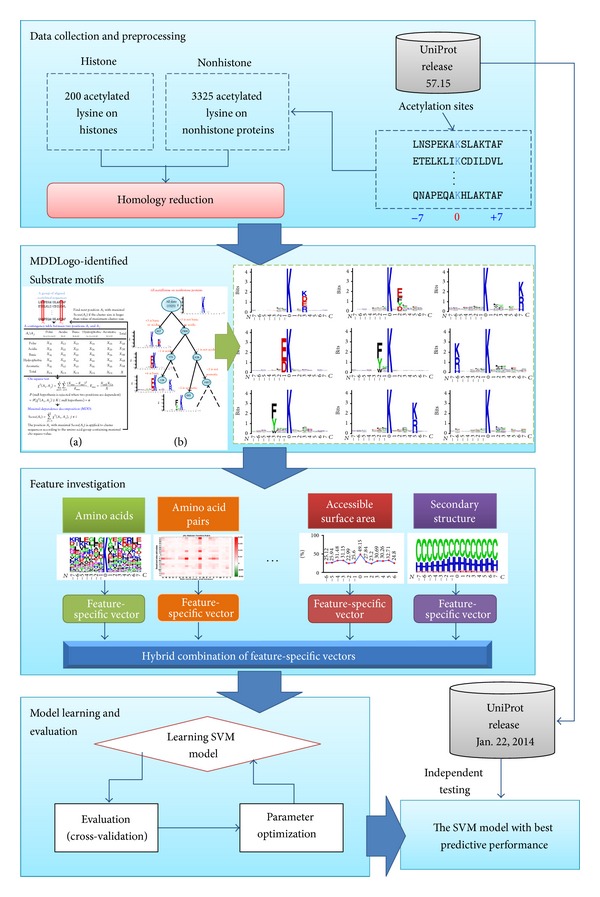
The conceptual diagram of AceK. The methodology of this study is composed of four major parts: data collection and preprocessing, substrate motif identification, feature investigation, and model learning and evaluation.

Here we applied LIBSVM [35] to implement the prediction model for discriminating acetylated lysine sites and nonacetylated lysine sites. A radio basis function (RBF) was adopted as the kernel function. Gamma determines the RBF kernel function, and cost controls the hyperplane softness. Gamma parameter, which determines the RBF kernel function, and cost parameter, which controls the hyperplane softness, were tuned to yield the best performance. Prior to the construction of a final model, the predictive performance of models using different features was evaluated by performing fivefold cross-validation. Firstly, the training data was divided into five groups by splitting each dataset into five approximately equal sized subgroups (Figure 2). During cross-validation, one subgroup was regarded as the test set, and the remaining four subgroups were regarded as the training set. Cross-validation was repeated for five rounds, where each subgroup is used as a test set once. The validation results were then combined to produce a single estimation. The advantage of performing a cross-validation evaluation is that all original data are regarded as both training set and testing set, and each dataset is used for testing exactly once [36].

Specificity (SP), sensitivity (SN), precision (PRE), accuracy (ACC), and Matthew's correlation coefficient (MCC) are utilized to evaluate the performance of classification. They are defined as follows:

 sensitivity (Sn) = TP/(TP + FN),
 specificity (Sp) = TN/(TN + FP),
 accuracy [37] = (TP + TN)/(TP + FP + TN + FN),
 Matthew's correlation coefficient (MCC) = (((TP × TN) − (FN × FP))
 
$\;/\sqrt{\left(\text{TP}+\text{FN}\right)\times \left(\text{TN}+\text{FP}\right)\times \left(\text{TP}+\text{FP}\right)\times \left(\text{TN}+\text{FN}\right)})$,

where TP, TN, FP, and FN are the numbers of true positives, true negatives, false positives, and false negatives [38]. The value of MCC is one for a perfect prediction, zero for a completely random prediction, and −1 for a perfectly inverse correlation.

## 3. Results and Discussion

### 3.1. Amino Acid Composition Analysis

**Figure 3:**
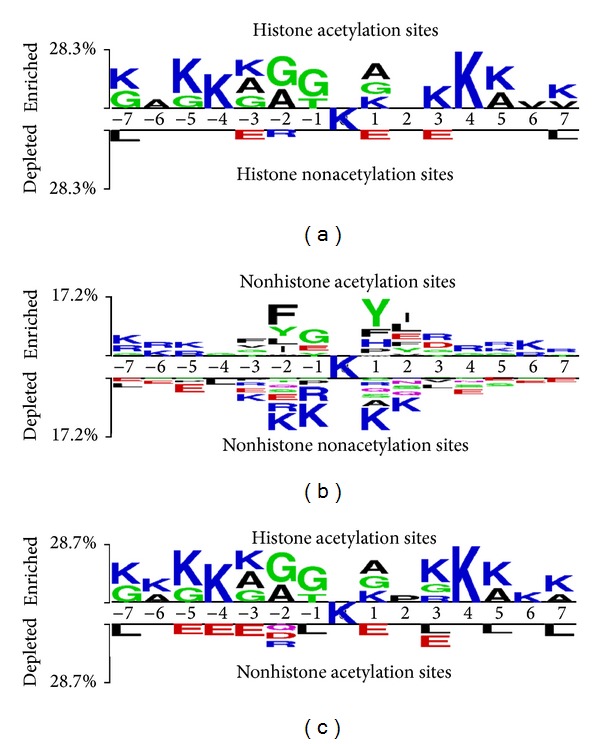
Two Sample Logo presents the compositional biases of amino acids around acetylation sites compared to the nonacetylation sites on histone and nonhistone sets. The significant amino acids around acetylated lysine residue are enriched from the positive dataset and presented in upper panel (*P* < 0.01). Relatively, the high frequency of amino acids around nonacetylated lysine is depleted from the negative dataset and presented in lower panel.

A web-based tool, Two Sample Logo [39], was used to graphically represent the sequence conservation of proteins by detecting and displaying statistically significant differences in position-specific symbol compositions between two sets of multiple sequence alignments. Different residues at the same position were scaled according to their frequency. To investigate the substrate specificity of acetylated lysine sites, WebLogo [26] was applied to generate the sequence logo of positive training dataset presenting with 15-mer flanking sequences. Two Sample Logo revealed that the most pronounced feature of acetylation sites in histones was the abundance of charged amino acids, especially the positively charged lysine (K) at positions −7, −5 ~ −3, +1, and +3~+7, glycine (G) at positions −7, −5, −3 ~ −1, and +1, alanine (A) at positions −6, −3, −2, +1, and +5 (*P* < 0.01, upper panel of Figure 3(a)). The feature is significantly different between nonacetylation sites on histones, which contain negatively charged amino acids glutamate (E) at positions −3, +1, and +3 and leucine (L) at −7 and +7 (*P* < 0.01, lower panel of Figure 3(a)). Regarding the feature of nonhistone proteins, fewer noticeable amino acids groups were displayed in acetylation set compared to that on histone proteins (upper panel of Figure 3(b)). However, more positively charged amino acids (e.g. K/R) were found to be located in distinct sequences. This is in contrast to K/R residues found to be located around nonacetylated lysines (lower panel of Figure 3(b)). In addition, amino acid composition around lysine acetylation sites between histones and nonhistone proteins presented that K, G, and A are more abundant in histone. However, negatively charged amino acids D/E and the hydrophobic amino acid L are more abundant in nonhistone protein. Results show that there exist different patterns surrounding lysine acetylation sites between histones and nonhistone proteins. This information may be helpful for identifying lysine acetylation sites. Therefore, we constructed different prediction models for histone and nonhistone proteins.

### 3.2. Performance of Fivefold Cross-Validation

Two prediction models, histone model and nonhistone model, were developed by using histones and nonhistone proteins. Various SVM models were built with different features (20D binary code, BLOSUM62, AAC, AAPC, ASA, PWM, and PSSM) and their combinations. SVM models were evaluated by 30 runs of fivefold cross-validation, and the best performance model was adopted as the final prediction model. In single feature models, the AAPC shows the best performance, which achieves accuracy of 0.8 and MCC of 0.483 in histones (Table 3). Relatively, BLOSUM62 presents the best performance in nonhistone proteins achieving sensitivity (Sn), specificity (Sp), accuracy (Acc), and MCC of 0.697, 0.723, 0.712, and 0.375, respectively. In addition to histone and nonhistone models, a mixed model was also generated by the use of both histones and nonhistone proteins. Using mixed analysis of BLOSUM62 and AAPC, the performance model in nonhistone proteins was improved to achieve a sensitivity of 0.706 and accuracy of 0.715 (Table 3). This demonstrates that our method is helpful and effective for identifying lysine acetylation sites by classifying protein into histone and nonhistone.

### 3.3. MDD-Detected Substrate Motifs for Lysine Acetylation Sites

To improve the detection of the conserved motifs from a large-scale acetylation dataset, we further applied the maximal dependence decomposition (MDD) to cluster all 200 and 3325 experimental acetylated peptide sequences in histone and nonhistone protein sets, respectively. Here, five subgroups of histone acetylation motifs and nine subgroups of nonhistone acetylation motifs can be captured exhibiting the most significant dependencies of amino acid composition between specific positions (Tables 4 and 5).

In the analysis of histone acetylation, we evaluated all of the acetylation sites and these 5 MDDLogo-clustered subgroups for their predictive performance by fivefold cross-validation. As shown in Table 4, H1 subgroup, which had conserved polar amino acids G/S/T/N/Q at position −1 and hydrophobic amino acids A/L/V/I/M/P at position +2, contained the highest predictive power at 0.930, 0.944, 0.942, and 0.812 for sensitivity, specificity, accuracy, and MCC, respectively. This motif was consistent with previous literature by structural study that G-AcK-X-P motif on histone H3 can be recognized by Tetrahymena GCN5 that is homologous to human acetylase P/CAF [40]. In addition, H3 motif, presenting known K-X_3_-AcK-X_3_-K motif at positions ±4 in our study, was highly consistent with the proteomic study for lysine acetylation of histone that displayed as the nuclear sequence motif for lysine acetylation of histones [7, 41]. Similar result also presented in H2 motif that higher frequency of K/R/H at the position +3 located in nucleus. However, two motifs in this study were not reported, suggesting they were novel motifs for lysine acetylation of histone.

In the analysis of nonhistone acetylation, 6 of 9 MDDLogo-clustered subgroups had the conserved motifs of positively and negatively charged amino acids (K/R/H and D/E) at a specific position. In particular, 817 acetylated peptides in the nH1 subgroup had positively and negatively charged amino acids on conserved motifs at the same specific positions. We evaluated all of the acetylation sites and these 9 MDDLogo-clustered subgroups for their predictive performance by fivefold cross-validation. As shown in Table 5, nH5 subgroup, which had a conserved F/Y/W at position −3, contained the highest predictive power at 0.934, 0.964, 0.956, and 0.885 for sensitivity, specificity, accuracy, and MCC, respectively. The MDDLogo-extracted cluster showed a higher performance than the combined MDDLogo-clustered motifs, which achieved a sensitivity, specificity, accuracy, and MCC of 0.863, 0.885, 0.88, and 0.706, respectively. This analysis indicates that the acetylated sequences in a large-scale dataset can be alternatively clustered by MDD method in order to significantly enhance the signal of amino acids motif and improve the performance of the predictive model.

Similar to nH5 subgroup, nH4 motif from nonhistone dataset also contained a conserved F/W/Y at the position −2 and Y at the position +1 flanking on the acetylated lysines that had comprehensively identified the acetylome in different sublocalization fractions and presented the distribution in nucleus and cytoplasm [5]. In addition, our analyzed data was also highly consistent with the previous studies [41], including the nH1 motif that higher frequency of K/D/E/R at the position +3 located in nucleus, cytoplasm, mitochondria, and ER-Golgi, nH2 motif (F/V at position +2 in mitochondria), nH3 motif (D/E at position −1 in cytoplasm and mitochondria), nH6 motif (K/R at position +5 in nucleus and ER-Golgi), and nH8 motif (K/R at position −6 in nucleus and ER-Golgi). We also found that KXE motif was consistent with previous studies in nonhistone proteins and nH2 motif was KXE motif (E/D/F/Y/W at position +2) in Table 5. In addition, two motifs in this study were not reported, suggesting that there were novel motifs for lysine acetylation of* non*histone. Therefore, this study can help us to find out some potential motifs for regulation of protein localization.

### 3.4. Independent Testing and Comparison with Previous Approaches

**Figure 4:**
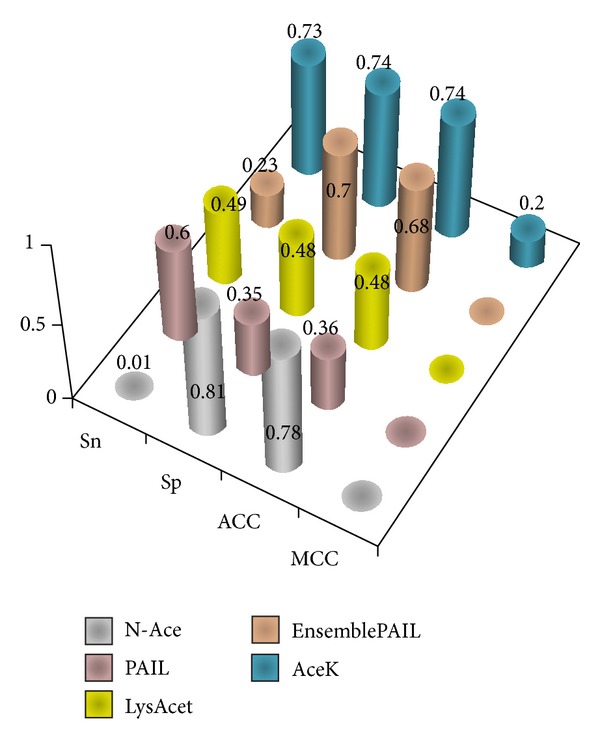
Comparison of independent testing performance between our method and four available online acetylation site prediction tools. Independent testing reveals that AceK outperforms the four available online acetylation site prediction tools with sensitivity (Sn) of 0.73, specificity (Sp) of 0.74, accuracy (ACC) of 0.74, and Matthew's correlation coefficient (MCC) of 0.2.

The independent dataset generated from UniProtKB/Swiss-Prot was used to evaluate our prediction models and was also employed to evaluate previous approaches. Using all acetylation sites in the independent dataset, the performance of AceK achieved a sensitivity of 0.73, a specificity of 0.74, an accuracy of 0.74, and the MCC of 0.20 (Figure 4). To further demonstrate the effectiveness of our method, the independent testing set was also used to compare our method with 4 other published websites for acetylation prediction: N-Ace, PAIL, LysAcet, and EnsemblePAIL. The result indicates that the prediction power yielded by our method was superior to that by the other 4 prediction tools, especially in sensitivity and MCC.

### 3.5. Implementation of Web Server

**Figure 5:**
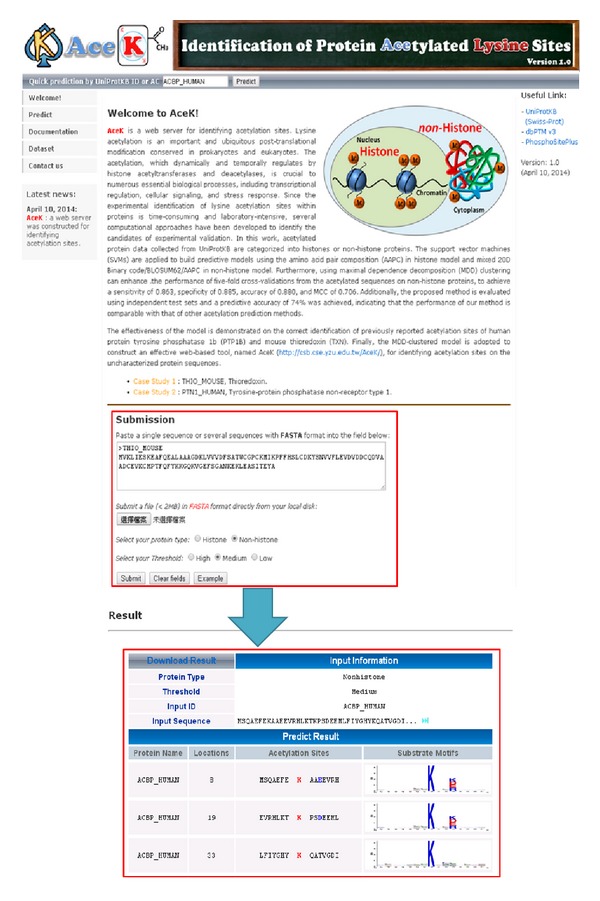
AceK web interface. The AceK website allows users to submit a single or several sequences in FASTA format and responds with a results page containing the downloadable predicted results.

A web server, AceK, was constructed for identifying the lysine acetylation sites on histones and nonhistone proteins. For this implementation, AAPC feature was utilized for the histone model, and mixed features between BLOSUM62 and AAPC were employed for the nonhistone model. As shown in Figure 5, AceK web server provides user-friendly interface and prediction results page. Users can submit the protein sequence in FASTA format and select a protein type for identifying potential lysine acetylation sites. The independent testing dataset used in our study is also provided on the website. The web server is available at http://csb.cse.yzu.edu.tw/AceK/.

## 4. Conclusion

This is the first study to identify the potential lysine acetylation sites on histone and nonhistone proteins. We not only demonstrated that the histone and nonhistone models had better predictive performances than the mixed model but also showed that our models exhibit significantly improved prediction sensitivity, specificity, accuracy, and MCC of lysine acetylation sites compared to previous approaches. Using AAC of flanking regions of lysine acetylation sites, we found that highly conserved sequence existed among histones, enriched with positively charged amino acids in distinct location. However, the negatively charged amino acids are more abundant surrounding acetylation sites of nonhistone proteins. A Two Sample Logo was constructed to display compositional biases between acetylation and nonacetylation sites in histone and nonhistone model. MDDLogo-clustered method also enhanced the performances of fivefold cross-validations compared to all acetylation sites without grouping. In addition, AceK web server was developed using the SVM models with AAPC feature for the histone model, BLOSUM62, and AAPC features for the nonhistone model. In this study, we provide reliable predictions and characterize the various features of these lysine acetylation sites of both histone and nonhistone proteins. We believe that this method will be helpful in identifying lysine acetylation and could be extended to provide the further information for the substrate specificity of nonhistone proteins in different subcellular localizations in the future.

## Figures and Tables

**Table 1:** Data statistics of training set and independent testing set.

Data resource			Acetylation sites (positive data)	Nonacetylation sites (negative data)
Training set	UniProtKB (v5715)	Histone	200	2729
		Nonhistone	3325	72445

Independent testing set	UniProtKB (January 22, 2014)	Histone	9	93
		Nonhistone	66	1737
		Combined nonredundant dataset	**75**	**1830**

**Table 2:** The amino acids group of MDDLogo used in this study.

Chemical properties	Amino acids
Polar group	Glycine (G), serine (S), threonine (T), cysteine (C), glutamine (Q), and asparagine (N)
Acidic group	Aspartic acid (D) and glutamic acid (E)
Basic group	Lysine (K), arginine (R), and histidine (H)
Hydrophobic group	Alanine (A), valine (V), leucine (L), isoleucine (I), proline (P), and methionine (M)
Aromatic group	Phenylalanine (F), tyrosine (Y), and tryptophan (W)

**Table 3:** Fivefold cross-validation results on histone and nonhistone model trained with various features.

Dataset	Training features	Sn	Sp	Acc	MCC
Histone	20D binary code	0.725	0.743	0.740	0.370
	BLOSUM62	0.745	0.758	0.756	0.400
	Amino acid composition (AAC)	0.750	0.761	0.386	0.407
	Amino acid pair composition (AAPC)	**0.790**	**0.802**	**0.800**	**0.483**
	Accessible surface area (ASA)	0.645	0.663	0.660	0.236
	Position weight matrix (PWM)	0.700	0.721	0.718	0.329
	Position-specific scoring matrix (PSSM)	0.710	0.724	0.721	0.339

Nonhistone	20D binary code	0.698	0.714	0.710	0.366
	BLOSUM62	0.697	0.723	0.712	0.375
	Amino acid composition (AAC)	0.619	0.640	0.635	0.226
	Amino acid pair composition (AAPC)	0.628	0.660	0.652	0.253
	Accessible surface area (ASA)	0.562	0.620	0.605	0.159
	Position weight matrix (PWM)	0.606	0.625	0.602	0.200
	Position-specific scoring matrix (PSSM)	0.665	0.695	0.688	0.319
	BLOSUM62 + AAPC	**0.706**	**0.718**	**0.715**	**0.377**

A total of 3525 lysine sequences were applied in positive and negative data. Sn, sensitivity; Sp, specificity; Acc, accuracy; MCC, Matthew's correlation coefficient.

**Table 4:** The five MDDLogo-clustered subgroups and their performances of fivefold cross-validations from 200 acetylation sites in histone dataset.

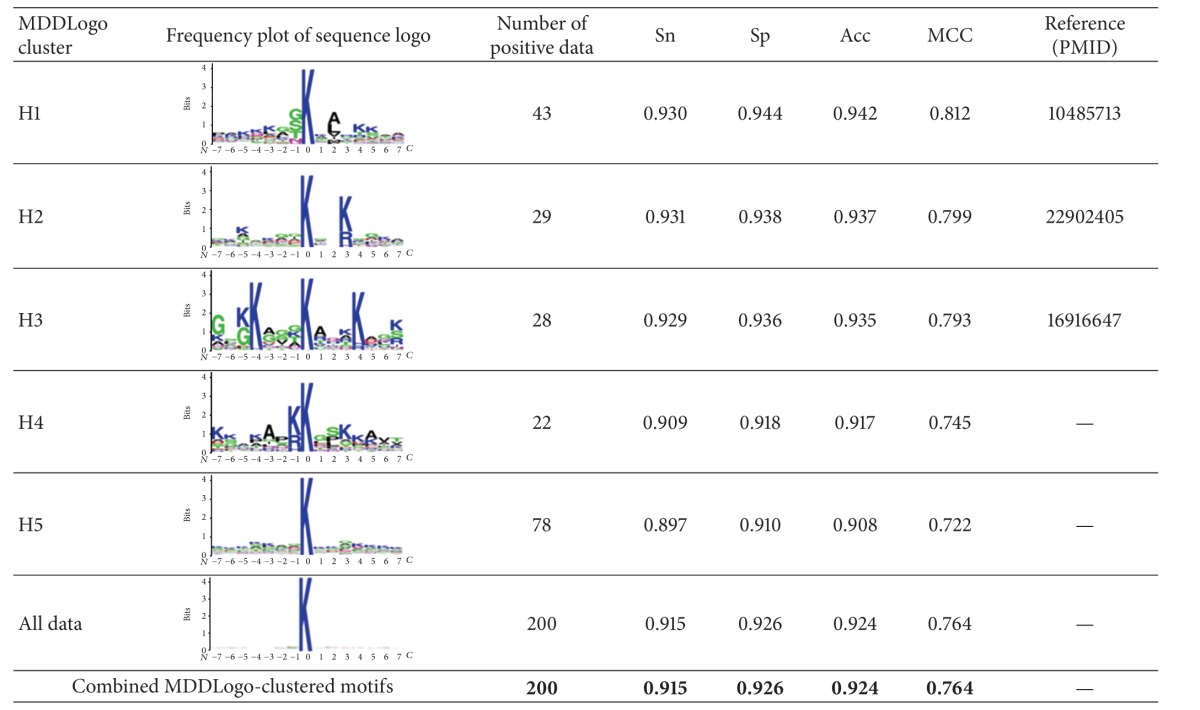

Sn, sensitivity; Sp, specificity; Acc, accuracy; MCC, Matthew's correlation coefficient.

**Table 5:** The nine MDDLogo-clustered subgroups and their performances of fivefold cross-validations from 3325 acetylation sites in nonhistone dataset.

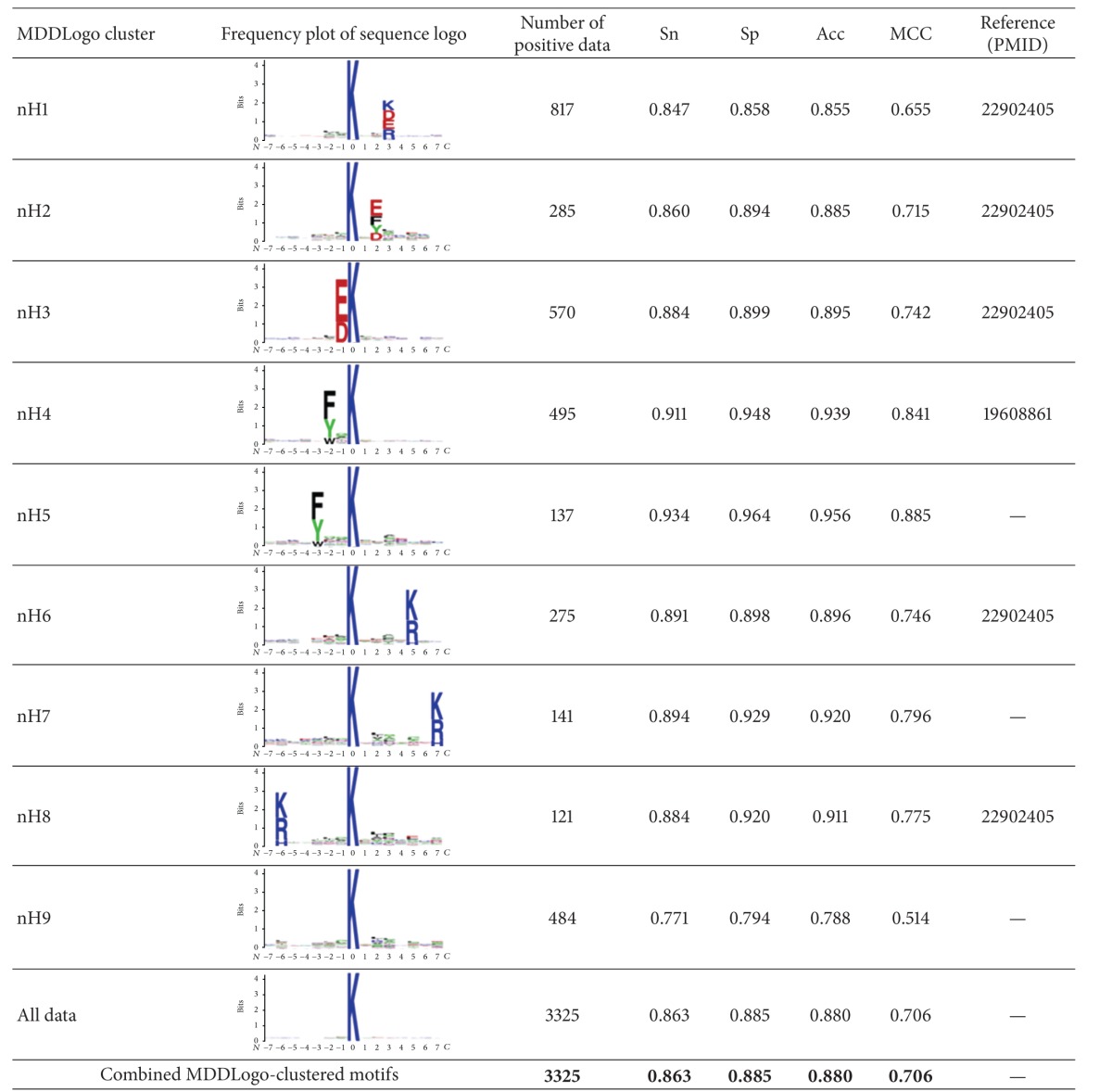

Sn, sensitivity; Sp, specificity; Acc, accuracy; MCC, Matthew's correlation coefficient.

## Availability

The proposed method is implemented as a web-based resource, which is now freely available to all interested users at http://csb.cse.yzu.edu.tw/AceK/. All of the datasets used in this work are also available for download on the website.
